# Telemedizin und KI-gestützte Diagnostik im Alltag der Viszeralmedizin

**DOI:** 10.1007/s00104-024-02213-8

**Published:** 2024-12-30

**Authors:** Matthias Grade, Verena Uslar

**Affiliations:** 1 Abteilung für Gastroenterologie, Allgemeine Innere Medizin und Infektiologie, Christliches Krankenhaus Quakenbrück GmbH, Lehrkrankenhaus der Carl von Ossietzky Universität Oldenburg und der European Medical School Oldenburg–Groningen (EMS), Danziger Straße 2, 49610 Quakenbrück, Deutschland; 2 Universitätsklinik für Viszeralchirurgie – Pius-Hospital Oldenburg, Universitätsmedizin Oldenburg, Oldenburg, Deutschland

**Keywords:** Augmentated Reality, Virtuel Reality, Koloskopie, Adenomdetektionsrate, Avatar, Augmented reality, Virtual reality, Colonoscopy, Adenoma detection rate, Avatar

## Abstract

Die Fortschritte von Augmented (AR) und Virtual Reality (VR) in der Telemedizin sind rasant. Eine über die Distanz verfügbare AR konnte beispielsweise bereits erfolgreich in der Krisenintervention, z. B. in Kriegsgebieten, eingesetzt werden. Vielversprechend erscheinen die Potenziale der Telemedizin auch in strukturschwachen Gebieten oder in der Hinzuschaltung von Expertinnen oder Experten in Notaufnahmesituationen. Der Einsatz von Avataren in der Telemedizin bedarf noch weiterer Forschung und Entwicklung, um das Gefühl der Präsenz zu verbessern und damit die Akzeptanz zu erhöhen. Künstliche Intelligenz in der Endoskopie v. a. in der Koloskopie ist bereits in vielen gastroenterologischen Abteilungen gelebte tägliche Praxis. Über eine erhöhte Adenomdetektionsrate (ADR) ist der Benefit klar belegt. Studien konnten zudem eine im Vergleich zur Kontrollgruppe erhöhte Detektionsrate für sessile serratierte Adenome (SSA) und eine signifikant erhöhte Rate an dysplastischen Barrett-Arealen im oberen Gastrointestinal(GI)-Trakt (eventuelle Barrett-Karzinome) zeigen.

## Abstract (en)

Advances in telemedicine, exemplified by augmented reality (AR) and virtual reality (VR), are rapidly progressing. For instance, AR available over long distances has already been successfully utilized in crisis intervention, such as in war zones. The potential of telemedicine also appears promising in structurally weak areas or in the involvement of experts in emergency situations. Further research and development are needed on the avatars used in such telemedicine approaches to improve the sense of presence and thereby increase acceptance. Artificial intelligence (AI) in endoscopy, particularly in colonoscopy, is already a routine practice in many gastroenterology departments. The benefits are clearly evidenced by an increased adenoma detection rate (ADR). Studies have also shown a higher detection rate for sessile serrated adenomas (SSA) compared to the control group as well as a significantly increased rate of dysplastic Barrett’s areas in the upper gastrointestinal (GI) tract (potential Barrett’s carcinomas).

Die Telemedizin (TM) hat gerade in den letzten Jahren rasante Fortschritte gemacht, so auch in der Viszeralmedizin. Hologramme mit Darstellungen komplexer Pathologien können mit ExpertInnen, die als Avatare anwesend sind, interaktiv diskutiert und behandelt werden. Zusammen mit künstlicher Intelligenz (KI) kann sie in der gastroenterologischen Endoskopie eine Verbesserung der Detektabilität von Läsionen im Gastrointestinal(GI)-Trakt erzielen. So ermöglicht eine frühzeitige Entdeckung möglicher auffälliger Zellareale in der Endoskopie eine erfolgreiche Intervention, sei es endoskopisch oder auch operativ.

## Allgemeine Aspekte von KI und Telemedizin in der Viszeralmedizin

### Telemedizinische Historie

Anfänge der Telemedizin kennen wir weltweit bereits seit dem 19. Jahrhundert [1]. Eines der frühesten Beispiele stammt aus dem Jahr 1874 in der Gemeinde Barrow Creek/Australien. In dieser sehr entlegenen Gemeinde wurde mittels einer telegraphischen Nachricht medizinische Konsultation für eine Operation gesucht. Zudem konnte mittels telegraphischer Anweisungen eine Operation im 2000 km entfernten Perth erfolgreich durchgeführt werden. Auch im amerikanischen Bürgerkrieg 1861 bis 1865 wurden telegraphische Möglichkeiten genutzt, um Krankentransporte zu organisieren.

Der erste telemedizinische Notruf ist auf den Erfinder des Telefons, Alexander Graham Bell (1847–1922) selbst zurückzuführen, der bei einer Säureverletzung einen seiner Assistenten mittels Telefon um Hilfe rief. Die erste „Telefon-Auskultation“ fand wohl 1879 in den USA statt, hier bat der Arzt am anderen Ende der Leitung die Mutter, den Hörer dicht an die Brust des hustenden Kindes zu legen. [2].

Die erste „Telefon-Auskultation“ fand 1879 in den USA statt

Seit den 1920er-Jahren war hauptsächlich das Radio das Medium das v. a. für öffentliche Gesundheitsaspekte (Impfkampagnen etc.) genutzt wurde. Wurde die Videotelefonie bereits 1927 teilerfolgreich versucht, so ist ein Durchbruch dafür erst in den 1950er-Jahren geglückt. Ab 1955 wurden in Albany (New York) Vorlesungen über Funk für angehende Ärzte gehalten [3].

Einen Quantensprung stellt sicherlich die Teleradiologie dar, die ihre Anfänge bereits in den 1960er-Jahren hatte. Teleradiologische Systeme per se auf der Basis von Fernsehübertragungen sind jedoch seit den 1970er-Jahren v. a. in Frankreich und Schweden bekannt. Die Ärzte W. S. Andrus und T. K. Bird prägten den eigentlichen Begriff „Teleradiologie“ 1972 [4].

Werden Telegraphie, Telefonie und auch das Fernsehen als erste telemedizinische Generation angesehen, so folgt seit den 1970er-Jahren mit Einführung des Computers durch den deutschen Ingenieur Konrad Zuse und anderen [3, 5] die zweite telemedizinische Generation. Sie ist definiert durch Computer, Internet und World Wide Web. Seit den 1980er-Jahren sind z. B. durch den Bau von fünf Supercomputern zuerst in den USA lokale „vernetzte Räume“ entstanden. Parallel dazu wurden in anderen Ländern ähnliche vernetzte Räume generiert und damit die Grundlage des Internets geschaffen [3].

Neben den eigentlichen praktisch-klinisch orientierten Aspekten der Telemedizin (Teleradiologie etc.) ist durch die Bildung großer Datenbanken in der Medizin (Medline etc.) die Wissensgenerierung, gerade in der akademischen Welt (Forschungseinrichtungen, Universitäten etc.), geradezu ein Paradigmenwechsel eingetreten. War man früher darauf angewiesen die Bibliothek zur Öffnungszeit aufzusuchen, ist durch das Internet mit den Datenbanken ein Arbeiten jederzeit und an nahezu jedem Ort möglich. Entsprechende herunterzuladende Anwendungsprogramme (Apps) vervollständigen das Portfolio.

### Historische Bedeutung von KI in der Medizin

Erste Versuche mit künstlicher Intelligenz sind seit den 1950er-Jahren bekannt. Der Psychologe Frank Rosenblatt entwickelte ein erstes lernfähiges künstliches Neuron – er nannte es Perzeptron. Dieses einfache künstliche neuronale Netz gilt als Grundlage der heutigen KI. Inzwischen wird künstliche Intelligenz in vielen Bereichen der Medizin eingesetzt, von der Entwicklung von Medikamenten, über das Gesundheitsmonitoring bis hin zur Diagnostik und personalisierten bzw. chirurgischen Behandlung [6]. Besonders in der Diagnose von Brust- oder Hautkrebserkennung sind die Erfolge dabei klar erkennbar [7, 8].

Präsentiert wurde das erste System mit künstlicher Intelligenz für die Koloskopie durch die Firma Medtronic auf der United European Gastroenterology Week 2019 in Barcelona, Spanien. Als einer der ersten Endoskopiker, der bereits seit 2020 für die tägliche Koloskopieroutine KI einsetzt, gilt Prof. Oliver Pech, Chefarzt der Klinik für Gastroenterologie und interventionelle Endoskopie am Krankenhaus Barmherzige Brüder in Regensburg.

## Aktueller Stand von Telemedizin und KI

### Telemedizinische Aspekte 2024 in der Viszeralmedizin

Wenn man von telemedizinischen Aspekten in der Medizin allgemein sowie auch in der Viszeralmedizin spricht, müssen Systeme, die KI anwenden – also bis zu einem gewissen Maße selbst lernende Systeme –, von reinen telemedizinischen Tools klar abgegrenzt werden. Momentan klinisch einsatzbar sind dabei Systeme, die es ermöglichen, dass mehrere Personen sich in VR in einem Raum treffen und dort alle Befunde gemeinsam betrachten oder sogar manipulieren können und sich dabei gegenseitig als Avatare sehen. Mögliches Einsatzgebiet wäre hier z. B. das Tumorboard, wo sich z. B. externe Vorbehandelnde aktiver mit in die Diskussion einbringen können.

**Abb. 1:**
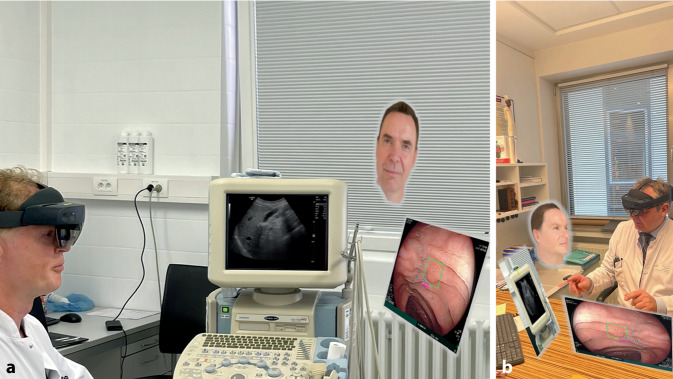
Telemedizin über eine Augmented-Reality-Brille. Diagnostische Maßnahmen (hier Ultraschall der Leber) können live aus dem Untersuchungsraum (**a**) aus Sicht des Untersuchers gestreamt werden, sodass das weitere Vorgehen direkt mit dem weiterbehandelnden Chirurgen (**b**) besprochen werden kann. Außerdem können vorherige Befunde (hier Endoskopie) von den Teilnehmern gemeinsam betrachtet und diskutiert werden und der jeweils andere Teilnehmer ist als interaktiver Avatar während der Konsultation im Display der Brille sichtbar

Augmented Reality kann z. B. schon sehr stabil dazu genutzt werden, innerhalb eines kleinen Personenkreises über große Entfernungen hinweg Befunde zu diskutieren und diagnostische Maßnahmen live aus der Sicht des aktuellen Behandlers mitzuverfolgen (Abb. 1). Ein weiterer bereits an der Universitätsklinik für Viszeralchirurgie am Pius-Hospital getesteter Anwendungsfall ist z. B. die Hinzuschaltung von ExpertInnen in eine aktuelle Notaufnahmesituation, sodass der Experte oder die Expertin aus dem Blickwinkel des gerade in der Notaufnahme anwesenden ärztlichen Personals den Patienten bzw. die Patientin sehen kann und so einen besseren Eindruck von der Gesamtsituation bekommt. So lässt sich, zumindest laut erster Rückmeldungen der Teilnehmenden, z. B. im Nachtdienst besser einschätzen, ob die Anwesenheit vor Ort notwendig ist oder nicht. Und auch die Hinzuschaltung in einen Operationssaal ist bereits erfolgreich erprobt worden, was perspektivisch dazu führen könnte, dass langwieriges Umziehen und Einwaschen eventuell entfallen können.

Voraussetzung für eine flächendeckende Anwendung ist in allen diesen Szenarien allerdings zum einen die Verwendung möglichst natürlicher Avatare, um den beteiligten Personen das Gefühl einer gemeinsamen, gleichberechtigten Diskussion geben zu können. Zum anderen sind telemedizinische VR- und AR-Funktionen sehr abhängig von einem gut funktionierenden Internet, welches momentan an den wenigsten Kliniken zu finden ist.

### KI in der Diagnostik

#### Aktueller Stand der Endoskopie

**Abb. 2:**
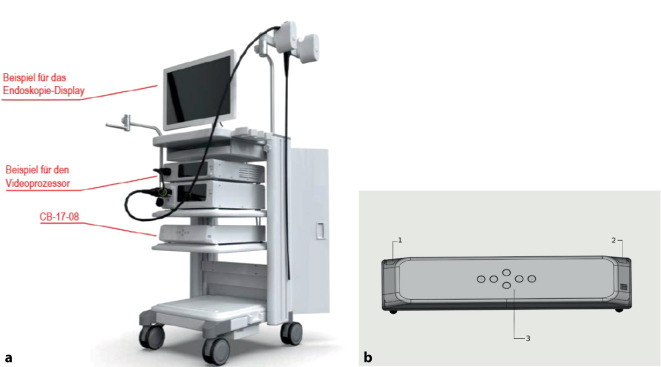
Darstellung des GI-Genius™ System CB1708-UN-01. (Mit freundlicher Genehmigung der Fa. Medtronic). **a** Marktüblicher Endoskopieturm; **b** KI-Prozessor

**Abb. 3:**
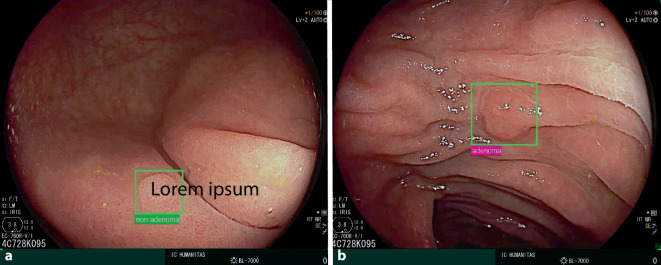
Bild eines hyperplastischen Polypen (**a**) und eines serratierten sessilen Adenoms (**b**) KI-unterstützt und augmentiert mittels GI-Genius™ System CB1708-UN-01. (© Dr. Grade; selbst am CQK durchgeführte Koloskopie)

Die Vorsorgekoloskopie wird in Deutschland als kassenärztliche Leistung seit dem 01.07.2019 für Frauen ab dem 55. und für Männer ab dem 50 Lebensjahr angeboten [9]. Kommerziell erhältliche Systeme (Abb. 2) können dabei mittels künstlicher Intelligenz eine erhöhte Rate an Adenomen, die sog. Adenomdetektionsrate (ADR), aufzeigen, speziell die der sog. serratierten sessilen Adenome (SSA, Abb. 3), und sind damit gemessen an der Effektivität der herkömmlichen Maßnahme (ohne KI) überlegen [10–12]. Allerdings wird von mehreren Autoren auf die zugrunde liegende endoskopische Kompetenz des Untersuchers als auch auf eine entsprechende ausreichende Vorbereitung des Patienten verwiesen [10].

Als Eckpfeiler der endoskopischen Diagnostik gelten die Spiegelung des oberen Gastrointestinaltraktes oder auch Ösophagogastroduodenoskopie sowie auch die Darmspiegelung oder auch Ileokoloskopie. Im Weiteren soll nun, unter anderem am Beispiel eigener Daten ausgeführt werden, inwieweit die KI in Deutschland in der Routinediagnostik bereits Einzug gehalten hat bzw.in welchem Bereich die Stärken sowie auch die Schwächen liegen.

#### Aktueller Stand der Koloskopie

Die Detektionsrate von Polypen (ADR) in der Ileokoloskopie, sei es aus der Vorsorgeindikation heraus oder auch unter kurativen Gesichtspunkten, unterliegt einer Reihe von Einschränkungen.

##### Als relevante Einschränkungen gelten:

- mangelnde Erfahrung des/der UntersucherIn,
- mangelnde Sauberkeit des Darmes,
- unzureichende Sedierung,
- zu kurze Koloskopierückzugszeit,
- technische Mängel wie mangelnde Hardware (Endoskope/Prozessoren etc.).

Auch unter den Voraussetzungen, dass eine entsprechend adäquate Reinigung des Darmes vorgenommen wurde, die technischen Geräte vorhanden sind und der geforderte Facharztstandard (oder gleichwertig) eingehalten wird, kann dennoch keine 100 %ige ADR garantiert werden.

Bereits seit längerem ist die sog. untersucherabhängige Größe oder auch „observer dependant bias“ (ODB) unabhängig vom Facharztstandard bekannt. Eine Zweituntersuchung oder auch Doppeluntersuchung [13–15] bzw. ein sog. „second look“ des rechten Hemikolons konnte signifikant die ADR verbessern [16], teilweise um bis zu 50 %.

Ein sog. „second look“ kann die Adenomdetektionsrate signifikant verbessern

Aktuell werden in Deutschland immer noch jährlich fast 60.000 kolorektale Karzinome gemeldet und 25.000 Todesfälle pro Jahr sind auf Darmkrebs zurückzuführen [17]. Seit Jahren ist daher die deutsche Gesellschaft für Verdauungs- und Stoffwechselkrankheiten (DGVS) bestrebt, i. S. von Leitlinien und Empfehlungen die Inzidenz kolorektaler Karzinome zu senken. Im Zeitraum von 2000 bis 2016 respektive bis 2018 sank nach Cardoso et al. 2021 die altersstandardisierte Inzidenz des kolorektalen Karzinoms (KRK) bei Männern um 22,4 % (65,3 vs. 50,7 pro 100.000) und bei Frauen um 25,5 % (42,7 vs. 31,8 pro 100.000; [17]). Die Mortalität reduzierte sich bei Männern um 35,8 % (29,6 vs. 19,0 pro 100.000) und um 40,5 % (19,0 vs. 11,3 pro 100.000) bei Frauen.

Dieses ist abgesehen von der häufigeren Inanspruchnahme einer Vorsorgekoloskopie sicherlich auch einer verstärkten Aufmerksamkeit und Sensibilität in der Gesellschaft bez. einer möglichen allgemeinen Krebsfrüherkennungdiagnostik (→ Vorsorgkoloskopie) geschuldet. Und auch die seit 1977 angebotene Untersuchung auf okkultes Blut im Stuhl könnte mitverantwortlich für diesen Rückgang sein. Und nicht zuletzt ist eine verbesserte Gerätetechnik und Bildverarbeitung (High Density, HD) verbunden mit sog. High-end-Endoskopen auch auf ambulanter Ebene (Facharztpraxen) zu bemerken.

#### KI in der Gastroskopie

In mehreren Studien ist der Benefit einer KI bei der Spiegelung des oberen Gastrointestinaltraktes klar belegt [18–22]. Gerade in der Detektabilität von Barrett-Läsionen konnte eine signifikante Verbesserung bez. des Erkennens dysplastischer Areale bis hin zu manifesten Karzinomen gezeigt werden [23, 24].

**Abb. 4:**
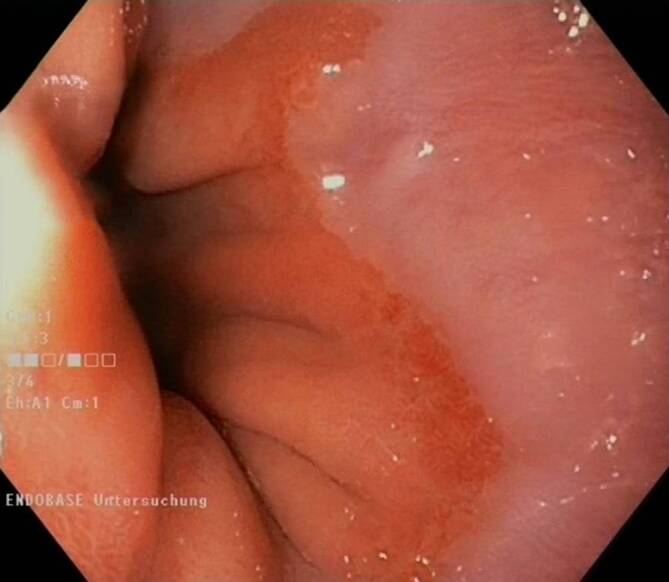
Typisches Bild eines Short-Barrett-Ösophagus mit hochgradig verdächtigen Dysplasiearealen (zukünftige „routinemäßige“ KI-Indikation). (© Dr. Grade, selbst am CQK durchgeführte Endoskopie)

Mit zur Hilfenahme virtueller als auch analoger Additive wie systemadaptierte chromoendoskopische Verstärker (NBI® etc.) als auch chromoendoskopische Verstärker wie Essigsäure oder auch Färbelösungen wie Toluidinblau etc. konnte eine weitere Zunahme von Dysplasiearealen (Abb. 4) gezeigt werden [25–28].

Aktuell ist jedoch in Deutschland kein KI-System für die Routinediagnostik verfügbar (Stand Juli 2024).

#### Rolle von KI in der täglichen Routinevorsorgekoloskopie

Neben der Anschaffung einer entsprechenden mit KI ausgerüsteten Endoskopieeinheit benötigt es zunächst keiner weiteren Maßnahme. Als Hardware wird das System (hier Genius CB 17–08) in der Nähe des jeweiligen Prozessors (das System kann mit jedem Prozessor unabhängig vom Hersteller betrieben werden, siehe Abb. 1) platziert und mit dem jeweiligen Bilderkennungssystem gekoppelt. Die Sinnhaftigkeit einer KI wird ersichtlich, wenn beim Erreichen des Zökalpoles der eigentliche Erkennungsprozess startet.

Nach dem Einschalten der KI erfolgt durch eine 100 %ige Sensitivität, erkennbar an farblich erkennbaren Rahmungen (Abb. 3), die Detektion möglicher Polypen. Eine Diagnose wird zudem durch ein darunterliegendes Feld vorgeschlagen (Adenoma bzw. NON-Adenoma). Der Untersucher entscheidet dann über eine konsekutive Intervention i. S. einer Polypektomie oder auch nicht, wenn z. B. Körner oder auch Stuhlreste fälschlicherweise als Polypen verkannt werden. Hier werden auch gleich sowohl die KI-assoziierten Stärken als auch Schwächen erkennbar.

Es besteht eine eindeutige Korrelation zwischen Koloskopierückzugszeit und Detektionsrate

Die Spezifität ist nach Studien bez. der Adenomerkennungsrate v. a. bei sessilen serratierten Adenomen (SSA) durchweg als überlegen beschrieben [16, 23, 29], wobei das Wesen der KI eben in einer kontinuierlich verbesserten ADR besteht. Dadurch unterscheidet sich dieses selbstlernende System eben von anderen z. B. auch technischen Hilfsmitteln wie virtuelle oder auch analoge chromoendoskopischen Strategien (z. B. NBI® etc. oder Färbelösung wie Essigsäure oder auch Blaufärbelösungen).

Eine mögliche Limitation des Systems liegt in der nicht adäquat eingehaltenen Koloskopierückzugszeit, die dem System die erforderliche Erkennungszeit, die, abhängig von der KI, zwischen einigen wenigen Sekunden und bis zu einer halben Minute liegt, nicht ermöglicht. Hier ist zu anmerken, dass nach einer großen Untersuchung aus der Mayo Klinik von 2006 eine eindeutige Korrelation zwischen adäquater Koloskopierückzugszeit (6–10 min ab Zökum) und der ADR per se besteht [29]. Einige Endoskopiker empfinden zudem die „flackernden“ Rahmen als störend.

In einer eigenen kleinen Fallserie wurden am Christlichen Krankenhaus Quakenbrück (CKQ) im Zeitraum Juni bis Juli 2024 bei jeweils 10 Patienten Vorsorgekoloskopien entweder ohne oder mit KI-Unterstützung durchgeführt. Die beiden Gruppen waren bez. Alter und Geschlecht und der Polypengröße vergleichbar (Tab. 1). Allerdings war die ADR in der Gruppe mit KI um 23 % höher (26 mit vs. 20 ohne KI) und der Grad der entdeckten High-grade-Dysplasien 3‑mal höher. Durch diese, wenn auch kleine Population, konnte in der KI-Gruppe somit die Zahl der potenziell risikobehafteten Polypen, eben Adenome (bis „high grade“), in einer höheren Detektionsrate gesehen und geborgen werden und damit eine potenzielle maligne Transformation frühzeitig verhindert werden.

**Tab. 1:** Falluntersuchung im Zeitraum Juni bis Juli 2024 am Christlichen Krankenhaus Quakenbrück

Vorsorgekoloskopie	Patientenalter 50–75 J weiblich/männlich (50:50)	Adenomdetektionsrate nach KI bzw. optischer Visualisierung	Polypengröße	Histologisches Ergebnis Intraepitheliale Dysplasie (R0 oder fraglich)
*Koloskopie n* *=* *10(CKQ 06/07 2024) Ohne KI*				
Colon ascendens distal	56 J, w	*N* = 2	5–8 mm	SSA, „low grade“
Colon ascendens proximal	50 J, m	*N* = 1	5–8 mm	A „high grade“, R0
Colon ascendens distal	69 J, w	*N* = 3	5–8 mm, N2 8–12, N1	Aa, „low grade“, R0
Colon transversum	59 J, w	*N* = 2	5–8 mm	A „low grade“, R0
Colon descendens	56 J, m	*N* = 1	8–12 mm	A „low grade“, R0
Colon descendens	65 J, w	*N* = 1	8–12 mm	HP
Colon sigmoideum	57 J, m	*N* = 2	5–8 mm, N1 8–12, N1	Aa „low grade“ R0
Colon sigmoideum	73 J, w	*N* = 2	15–20 mm N1 8–12 mm N1	Aa „low grade“ R0
Rektum	68 J, m	*N* = 3	5–8 mm	HP
Rektum	71 J, m	*N* = 3	5–8 mm	HP
*Koloskopie n* *=* *10 (CKQ 06/07 2024) mit KI *(GI-Genius™ System CB1708-UN-01)				
Colon ascendens proximal	55 J, w	*N* = 3	5–8 mm N2 8–12, N1	SSA, „low grade“, R0
Colon ascendens distal	59 J, m	*N* = 4	5–8 mm N3 8–12, N1	SSA, N3 „low grade“ R0 A N1 „low grade“ R0
Colon transversum	64 J, w	*N* = 3	8–12 mm	SSA, N2 „low grade“ R0 A N1 „high grade“ R0
Colon transversum	59 J, w	*N* = 3	5–8 mm	SSA, N3 „low grade“ R0
Colon descendens	64J, m	*N* = 2	8–12 mm N1 5–8 mm N1	SSA „low grade“ R0 HP
Colon sigmoideum	71 J, w	*N* = 2	5–8 mm N2	Aa N2 „low grade“ R0
Colon sigmoideum	57 J, m	*N* = 1	8–12 mm	A „high grade“ R0
Rektum	58 J, w	*N* = 4	5–8 mm N3 8–12 mm N1	A N3 „low grade“ R0 Aa N1 „low grade“ R0
Rektum	66 J, m	*N* = 3	8–12 mm N2 5–8 mm N1	Aa „low grade“ R0 HP
Rektum	73 J, m	*N* = 1	8–12 mm	A „high grade“ R0

*A, Aa* Adenoma, *CKQ* Christlichen Krankenhaus Quakenbrück, *HP* Hyperplasia,* J* Jahre*, KI* künstliche Intelligenz, *NA* NON-Adenoma, *NC* „non conclusive“,* SSA* sessiles serratiertes Adenom,* w/m* weiblich/männlich

## Diskussion

Inzwischen gibt es ein breites Anwendungsspektrum für KI in der Medizin [6]. In der Diagnostik wird KI bereits für Brustkrebs und Hautkrebs sehr erfolgreich eingesetzt. Außerdem gibt es KI für die Erkennung von Knochenbrüchen und Herzkreislauferkrankungen [30, 31]. In der onkologischen Therapie kann KI eingesetzt werden, um Patientinnen und Patienten zu identifizieren, die von Immuntherapien profitieren könnten [32]. Im Bereich der Gesundheitsüberwachung haben sich sowohl individuelle ambulante Systeme durchgesetzt (z. B. sog. Smartwatches oder Handy-Applikationen; [8]) als auch Systeme zum professionellen stationären Monitoring. Und auch in der Viszeralmedizin sind KI-basierte Systeme zwar vorhanden, aber teilweise noch nicht ausreichend evaluiert. Auch daher halten sich Skeptiker und Befürworter trotz bekannter Benefits immer noch die Waage [33]. Dies könnte vor allem daran liegen, dass es immer noch Ängste gibt, dass durch den verbreiteten Einsatz von KI Arbeitsplätze verloren gehen könnten [6]. Dem kann aber entgegengehalten werden, dass der Fachkräftemangel, der bereits in vielen Praxen und Kliniken deutlich spürbar ist, sich in den nächsten Jahren noch deutlich verstärken wird. Dies gilt nicht nur für die Primärversorgung [34], sondern für den gesamten Gesundheitssektor. So werden im Jahr 2035 nach einer Studie von PWC ca. 35 % aller Stellen unbesetzt sein [35].

Die bisher vorhandenen Studien zeigen, dass im Mittel ca. 20 % der relevanten Adenome in einer Koloskopie übersehen werden [36]. Daher ist es nicht verwunderlich, wenn unsere eigenen Daten bei der Anwendung von KI eine um mehr als 20 % erhöhte ADR zeigen und dieser Benefit auch in anderen Studien gezeigt werden konnte [23]. Speziell in der Endoskopie des oberen Gastrointestinaltraktes ist der unmittelbare Benefit in der Detektion auffälliger (dysplastischer) Zellareale, wie z. B. beim Barrett-Karzinom, offensichtlich. Eine verfügbare routinemäßige KI für den oberen GI gibt es jedoch leider bislang nicht (Stand 07/2024).

Weitere Forschung zu solchen Systemen und auch sicherlich flächendeckende Schulungen erscheinen sinnvoll, um die Akzeptanz solcher Systeme zu erhöhen. Hier ist das Beispiel der Vorsorgekoloskopie beim Kolonkarzinom das erste und auch beste Beispiel.

Allerdings wird hier gleichzeitig eine Schwäche im deutschen Gesndheitssystem offensichtlich. Gilt die Vorsorgekoloskopie als eine Leistung vornehmlich der ambulant tätigen Fachärzte, besonders der Gastroenterologen, ist neben einer ausreichenden apparativen, aber auch die personell flächige Abdeckung gerade im ländlichen Bereich unabdingbar.

In der Kombination mit AR oder VR kann die Telemedizin ihr komplettes Potenzial entfalten

Hier kommt die Telemedizin ins Spiel. Seit der ersten Fernauskultation eines hustenden Kindes mittels eines Telefons 1879 in einer Kleinstadt in den USA ist die telemedizinische Entwicklung rasant fortgeschritten.Über verstärkende Stethoskope bis hin zu avatarunterstützter hologrammbasierter Operationsführung oder Konsultation in nahezu jedem medizinischem Bereich sind der medizinischen Entwicklung anscheinend keine Grenzen gesetzt. Besonders in der Kombination mit AR oder VR kann die Telemedizin künftig ihr komplettes Potenzial entfalten. AR und VR mögen dabei immer noch ein wenig futuristisch anmuten. Nur ist diese Technologie bereits in vielen Operationsräumen, wie o. g., bereits angekommen und gelebte tägliche Realität.

Eine weitere nicht zu unterschätzende Stärke der AR und VR ist in der Aus- und Weiterbildung von Spezialisten (Ärzten aber auch Pflegepersonal) sowie der Konsultation zu sehen. Wie in einigen o. g. Studien gezeigt, ist erkennbar geworden, dass auch über große Entfernungen hinweg Schulungen sowohl im operativen als auch im endoskopischen, radiologischen oder konservativen Setting möglich sind. Gerade Länder des globalen Südens könnten hier wesentlich profitieren. Ausbildungsprogramme prägradual als auch postgradual z. B. i. R. von Medical Education erscheinen sinnvoll. Universitäten sind in der Pflicht z. B. über erweiterte modulare Lernformate diese Thematik zu vermitteln, damit sich die medizinische Versorgung auch in Zukunft weiterentwickeln kann.

## Fazit für die Praxis

- Der Einsatz künstlicher Intelligenz (KI) erfolgt bereits in vielen verschiedenen medizinischen Bereichen sehr erfolgreich, besonders in der Krebsdiagnostik.
- In der Viszeralmedizin ist die KI-gestützte Koloskopie ein sehr erfolgreiches Beispiel.
- Über telemedizinische Anwendungen können sich ExpertInnen auch über große Entfernungen konsultieren und interaktiv an Diagnose und Behandlung teilnehmen.
- In der Viszeralmedizin kann über die Telemedizin mit Avataren eine interdisziplinäre Behandlung von Patienten erfolgen.
- Sowohl KI als auch Telemedizin können dem Fachkräftemangel entgegenwirken.

## Abkürzungen

AD: Adenom
ADR: Adenomdetektionsrate
AI: Artificial intelligence
AR: Augmentated Reality
HP: Hyperplasie
MR: Mixed reality
NBI: Narrow band imaging
NC: Non conclusive
ODP: Observer dependent bias
SC: Standard colonoscopy
SSA: Sessiles serratiertes Adenom
VR: Virtual Reality
